# Emergency Department Visits for Circulatory System Diseases: A Study of Primary Diagnoses Using National Hospital Ambulatory Medical Care Survey (NHAMCS) Data From 2016 to 2022

**DOI:** 10.7759/cureus.83569

**Published:** 2025-05-06

**Authors:** Michael K Olanite, Bernard Wiredu, Alexander S Figueroa, Zainab Baye, Saluwa Nansimbi, Edidiong Enyeneokpon, Chinwe O Ayozie, Okelue E Okobi, Nneka Muoghalu

**Affiliations:** 1 Emergency Medicine, University Hospitals Dorset National Health Service (NHS) Foundation Trust, Bournemouth, GBR; 2 Internal Medicine/Oncology, Saint James School of Medicine, Park Ridge, USA; 3 Medicine, University of the East Ramon Magsaysay Memorial Medical Center (UERMMMC), Quezon City, PHL; 4 Medicine and Surgery, University of Lagos, Lagos, NGA; 5 Cardiology, Alberta Health Services, Calgary, CAN; 6 Acute Medicine, Pilgrim Hospital, Boston, GBR; 7 Internal Medicine/General Practice, Centra Clinic, Houston, USA; 8 Family Medicine, International Medical Graduate (IMG) Research Academy and Consulting, Homestead, USA; 9 Family Medicine, Larkin Community Hospital Palm Springs Campus, Miami, USA; 10 Family Medicine, Lakeside Medical Center, Belle Glade, USA; 11 Internal Medicine, University College Hospital, Ibadan, NGA

**Keywords:** circulatory system, emergency department, large database, nhamcs, retrospective study

## Abstract

Background: Circulatory system diseases are leading causes of emergency department (ED) visits in the United States. Understanding trends in these visits is crucial for healthcare planning.

Objective: This study aimed to identify the most frequent primary circulatory system diagnoses presenting to EDs, examine temporal trends in ED visit rates from 2016 to 2022, and assess disparities by patient age, sex, race/ethnicity, insurance status, and geographic region.

Materials and methods: A cross-sectional analysis of the National Hospital Ambulatory Medical Care Survey (NHAMCS) data (2016-2022) identified ED visits with a primary circulatory disease diagnosis using International Classification of Diseases (ICD) codes. Weighted estimates ensured national representativeness. Descriptive statistics and inferential statistics were used to assess trends. Analyses were performed in STATA 17 (StataCorp., College Station, TX, USA) (p<0.05).

Results: Overall ED visit rates for circulatory diseases rose from 15 per 1,000 in 2016 to 17 per 1,000 in 2019 (p<0.01), declined to 16 per 1,000 in 2020 (p<0.05 vs. 2019), and stabilized at 17 per 1,000 in 2021-2022 (no significant change, p=0.12). Essential hypertension (14%), hypertensive heart or kidney disease (13%), acute myocardial infarction (11%), atrial fibrillation/flutter (10%), and ischemic stroke (8%) represented the five most common primary diagnoses, accounting for over 56% of circulatory presentations. Older adults (65+) exhibited the highest visit rates (peaking at 55 per 1,000 in 2022), males had slightly higher rates than females (18 vs. 16 per 1,000 in 2022), and non-Hispanic Black individuals had persistently elevated rates (28 per 1,000 in 2022) compared to non-Hispanic White individuals (17 per 1,000) and Hispanic individuals (12 per 1,000) (all p<0.05). The Midwest and Northeast regions reported the highest rates, whereas the West had the lowest, underscoring geographic variation in circulatory disease burden. Medicare beneficiaries had the highest utilization (44 per 1,000 in 2022), highlighting insurance-related disparities.

Conclusions: In this nationally representative analysis, primary ED presentations for circulatory diseases were dominated by hypertension-related conditions and acute ischemic events. Stabilization of rates post-2020, particularly among older and Medicare-insured patients, indicates potential targets for intervention. Strengthening primary care access and preventive programs, especially for older adults and non-Hispanic Black individuals, may reduce ED reliance and improve equity in cardiovascular care.

## Introduction

Circulatory system diseases, including cardiovascular conditions such as ischemic heart disease, hypertensive emergencies, arrhythmias, and heart failure, as well as cerebrovascular events like ischemic and hemorrhagic stroke, remain a leading cause of emergency department (ED) visits worldwide [1]. These conditions, including ischemic heart disease, hypertensive emergencies, arrhythmias, stroke, and heart failure, often present with acute symptoms requiring immediate medical intervention [2-3]. The high burden of circulatory diseases in EDs reflects their significant morbidity and mortality, as well as their impact on healthcare resource utilization [4]. Understanding the primary diagnoses associated with these conditions in ED settings is crucial for optimizing patient management strategies, improving emergency response systems, and developing targeted prevention programs [5].

Cardiovascular diseases (CVDs) are the leading cause of death globally, accounting for approximately 17.9 million deaths annually, as reported by the World Health Organization [6]. In the United States, the Centers for Disease Control and Prevention estimates that over 800,000 individuals suffer from myocardial infarction each year, with nearly 20% of these events being silent [7]. Additionally, hypertensive crises and stroke contribute significantly to emergency healthcare encounters, particularly among older adults and individuals with preexisting conditions such as diabetes and obesity [8]. Compared to other acute presentations such as respiratory infections and traumatic injuries, circulatory diseases demonstrate persistently high ED utilization, often ranking among the top three reasons for emergency care visits, underscoring their unique resource demands [3-7]. Thus, individuals often seek emergency care services for cardiovascular conditions based on factors such as level of education, socioeconomic status, access to primary care services, and geographic location. It has been disclosed that persons with low incomes, including individuals with limited health literacy levels, tend to have higher rates of ED visits as a result of insufficient access to both preventive and outpatient cardiovascular care [7,8]. Such disparities are increasingly pronounced in economically disadvantaged rural and urban communities, where limited access to healthcare increases the requirement for emergency care for circulatory diseases [7,8].

Patients typically present with chest pain or pressure, shortness of breath, dizziness, sudden weakness or numbness (in cases of stroke), palpitations, or acute onset headache, symptoms that prompt immediate ED evaluation [2-3]. The pathophysiology of these conditions is primarily driven by atherosclerosis, a chronic inflammatory process characterized by endothelial dysfunction, lipid accumulation, and plaque formation in arterial walls [9-10]. Risk factors such as hypertension, diabetes, and obesity exacerbate endothelial damage, promoting the deposition of low-density lipoproteins and the recruitment of inflammatory cells. Over time, plaques may rupture, triggering thrombosis and leading to acute events such as myocardial infarction or ischemic stroke [11]. Hypertensive crises result from excessive vasoconstriction and endothelial injury, causing severe elevations in blood pressure that can lead to end-organ damage, including heart failure, kidney failure, or intracerebral hemorrhage [12]. Stroke occurs when cerebral blood flow is obstructed (ischemic stroke) or when blood vessels rupture (hemorrhagic stroke), leading to neuronal injury and loss of function [13]. Underlying conditions such as diabetes and obesity contribute to CVDs by promoting insulin resistance, chronic inflammation, and dyslipidemia. These mechanisms collectively increase cardiovascular risk, making early detection and intervention essential for preventing complications and reducing mortality associated with circulatory system diseases [2-3].

The National Hospital Ambulatory Medical Care Survey (NHAMCS) is a comprehensive database that collects nationwide data on ED visits in the United States [14]. Conducted annually by the National Center for Health Statistics (NCHS), NHAMCS provides valuable insights into patient demographics, clinical presentations, diagnostic trends, and treatment patterns across various healthcare settings [14]. By analyzing NHAMCS data from 2016 to 2022, this study aims to identify the most common circulatory system diagnoses encountered in EDs, assess trends over time, and evaluate factors influencing the prevalence of these conditions [14].

Prior NHAMCS-based analyses have examined overall cardiovascular ED volumes and individual diagnoses such as myocardial infarction or stroke. However, few have simultaneously mapped the full spectrum of primary circulatory diagnoses alongside demographic and insurance disparities. This study aims to examine trends and disparities in ED visits for circulatory system diseases in the United States from 2016 to 2022, focusing on racial and ethnic differences. Our study’s novel contribution is twofold: (1) reporting the five most common primary circulatory diagnoses in a single, nationally representative analysis and (2) quantifying post‑COVID‑19 shifts and stabilization patterns across demographic, insurance, and geographic subpopulations. Emerging evidence indicates that ED utilization for acute conditions dipped sharply in 2020 before rebounding in 2021-2022 as deferred care resumed.

## Materials and methods

Data source and study design

This is a descriptive, cross-sectional analysis of NHAMCS data (2016-2022) [14]. NHAMCS is a nationally representative survey conducted by the National Center for Health Statistics (NCHS) to assess ambulatory care services in hospital EDs across the United States [14]. This cross-sectional study examined ED visits for circulatory system diseases, focusing on primary diagnoses (ICD‑10 I00-I99, including I10, I21, I48, and I60-I69).

Study participants and questionnaires

The study included all ED visits recorded in NHAMCS with a primary diagnosis of circulatory system disease. Participants were not individual patients but ED visits, as NHAMCS collects encounter-level data rather than longitudinal patient records [14]. NHAMCS employs structured questionnaires and standardized coding procedures to capture demographic details, visit characteristics, and primary diagnoses, ensuring consistency and comparability across survey years.

Data collection and quality assurance

NHAMCS data were collected through systematic sampling of hospital ED records. Trained abstractors recorded visit details, including patient demographics, clinical diagnoses, and disposition. The NCHS implemented rigorous data validation and quality control measures to ensure accuracy and reliability. Data were reviewed for completeness, coding consistency, and adherence to NHAMCS methodological standards before public release.

Variables of interest

The primary variable of interest was the ED visit rate for circulatory system diseases, classified according to ICD codes. Circulatory diseases were identified using ICD-10 codes I00-I99, encompassing essential hypertension (I10), acute myocardial infarction (I21), atrial fibrillation (I48), and stroke (I60-I69). Additional variables included patient demographic data, including age (categorized into three groups as 18-44 years, 45-64 years, and 65 years or older), gender (male/female), and race/ethnicity (non-Hispanic White individuals, non-Hispanic Black individuals, and Hispanic individuals). Socioeconomic factors, such as primary payment source (private insurance, Medicare, Medicaid, uninsured), geographic region (Northeast, Midwest, South, and West), and location of residence, such as metropolitan statistical areas (MSAs). The study also examined temporal trends across survey years to assess variations in ED utilization patterns. Comorbid conditions were excluded from regression models to focus on primary diagnosis drivers and avoid collinearity with age and insurance variables.

Data analysis and statistical methods

Descriptive statistics were used to summarize patient and visit characteristics. Weighted estimates were calculated using NHAMCS-provided survey weights to account for the complex sampling design and ensure national representativeness. Trends in ED visit rates were analyzed using chi-square tests for categorical variables and t-tests for continuous variables to examine associations between key demographic factors and ED visit rates for circulatory system diseases. Statistical significance was set at p<0.05, and analyses were conducted using STATA version 17 (StataCorp, College Station, TX, USA).

Ethical considerations

No institutional review board approval was required because the NHAMCS dataset is publicly available and de‑identified. The study adhered to ethical research guidelines, ensuring patient confidentiality and data integrity were maintained throughout the analysis.

## Results

An estimated 4.9 million ED visits (15 per 1,000 people; 95% CI: 12-18 per 1,000) occurred in 2016-2017, rising to 5.6 million visits (17 per 1,000; 95% CI: 14-19 per 1,000) in 2019, declining to 5.2 million visits (16 per 1,000; 95% CI: 13-20 per 1,000) in 2020, and stabilizing at 5.6 million visits annually (17 per 1,000; 95% CI: 14-20 per 1,000) in 2021-2022. A slight drop to 16 occurred in 2020 (95% CI: 13-20), followed by a rise to 17 in 2021-2022 (95% CI: 14-20). The wide confidence intervals reflect NHAMCS sampling variability due to smaller subgroup sample sizes, which can widen uncertainty bounds for some years. These fluctuations suggest notable increases in 2019 and post-pandemic years. Table 1 below details demographic, regional, and insurance trends in ED visits for circulatory diseases from 2016 to 2022.

**Table 1:** ED visit trends for circulatory system diseases within the period of 2016 to 2022 Data suppressed per NCHS reliability criteria (cell n<30 or relative standard error >30%) MSA: metropolitan statistical area, ED: emergency department, NCHS: National Center for Health Statistics

Characteristics	2016	2017	2018	2019	2020	2021	2022	Statistical test value	p-value
Overall visit rate (per 1,000 people)	15 (12–18)	15 (12–18)	14 (12–16)	17 (14–19)	16 (13–20)	17 (14–20)	17 (14–20)	-	-
Sex									
Female	15 (11–18)	14 (11–17)	14 (11–16)	16 (13–19)	15 (11–19)	16 (13–20)	16 (12–19)	t critical two-tail=2.44	0.02
Male	15 (12–18)	16 (11–20)	14 (12–17)	17 (14–21)	18 (13–22)	17 (13–20)	18 (14–22)		
Age group									
18-44 years	5 (3–6)	5 (4–7)	7 (5–9)	7 (5–9)	7 (4–9)	7 (5–10)	5 (3–6)	F critical=3.88, F value=300.5	<0.05
45-64 years	19 (15–23)	18 (13–23)	18 (14–22)	22 (17–26)	22 (15–28)	25 (18–31)	22 (16–27)		
65+ years	52 (39–65)	50 (36–63)	41 (34–48)	50 (43–58)	49 (36–62)	44 (34–54)	55 (43–67)		
Race/ethnicity									
Non-Hispanic White	17 (13–21)	15 (12–19)	14 (11–16)	18 (15–22)	19 (14–24)	19 (15–23)	17 (14–21)	F critical=3.88, F value=82.02	<0.05
Non-Hispanic Black	23 (15–31)	30 (18–42)	31 (24–37)	27 (20–34)	22 (12–31)	28 (19–37)	28 (19–37)		
Hispanic	8 (4–11)	6 (4–9)	8 (4–12)	9 (6–13)	8 (4–12)	8 (5–10)	12 (7–16)		
Region									
Northeast	17 (10–23)	15 (11-19)	11 (8–15)	14 (11–18)	18 (9–27)	16 (10–22)	18 (12–25)	F critical=3.15, F value=3.69	0.03
Midwest	19 (10–27)	20 (12–27)	15 (10–20)	15 (11–19)	17 (10-23)	19 (13–24)	16 (11–22)		
South	13 (9–17)	18 (12–24)	15 (12–19)	20 (16–25)	18 (11–24)	16 (10–23)	17 (12–22)		
West	13 (9–18)	9 (4–13)	12 (9–17)	14 (8–20)	16 (8–24)	15 (11–20)	16 (11–21)		
MSA									
MSA	13 (11–16)	15 (11–18)	14 (12–16)	15 (12–18)	-	-	-	t critical two-tail=4.3	0.38
Non-MSA	-	15 (10–21)	14 (7–22)	26 (17–35)	-	-	-		
Primary payment source									
Private insurance	5 (4–7)	6 (4–8)	5 (4–7)	7 (5–9)	6 (4–8)	8 (6–11)	6 (4–8)	F critical=3.88, F value=355	<0.05
Medicare	43 (33–53)	42 (30–54)	35 (29–42)	45 (38–52)	42 (32–52)	36 (28–44)	44 (35–54)		
Medicaid	13 (9–17)	15 (10–20)	14 (10–18)	13 (9–17)	16 (10–22)	16 (11–21)	17 (12–22)		
Uninsured	-	-	9 (5–13)	8 (5–12)	-	-	-		

Based on sex

Among females, ED visits numbered 2.3 million in 2016 (15 per 1,000; 95% CI: 11-18), 2.2 million in 2017-2018 (14 per 1,000; 95% CI: 11-17, 11-16), 2.5 million in 2019 (16 per 1,000; 95% CI: 13-19), 2.4 million in 2020 (15 per 1,000; 95% CI: 11-19), and 2.6 million in 2021-2022 (16 per 1,000; 95% CI: 13-20, 12-19).

**Figure 1:**
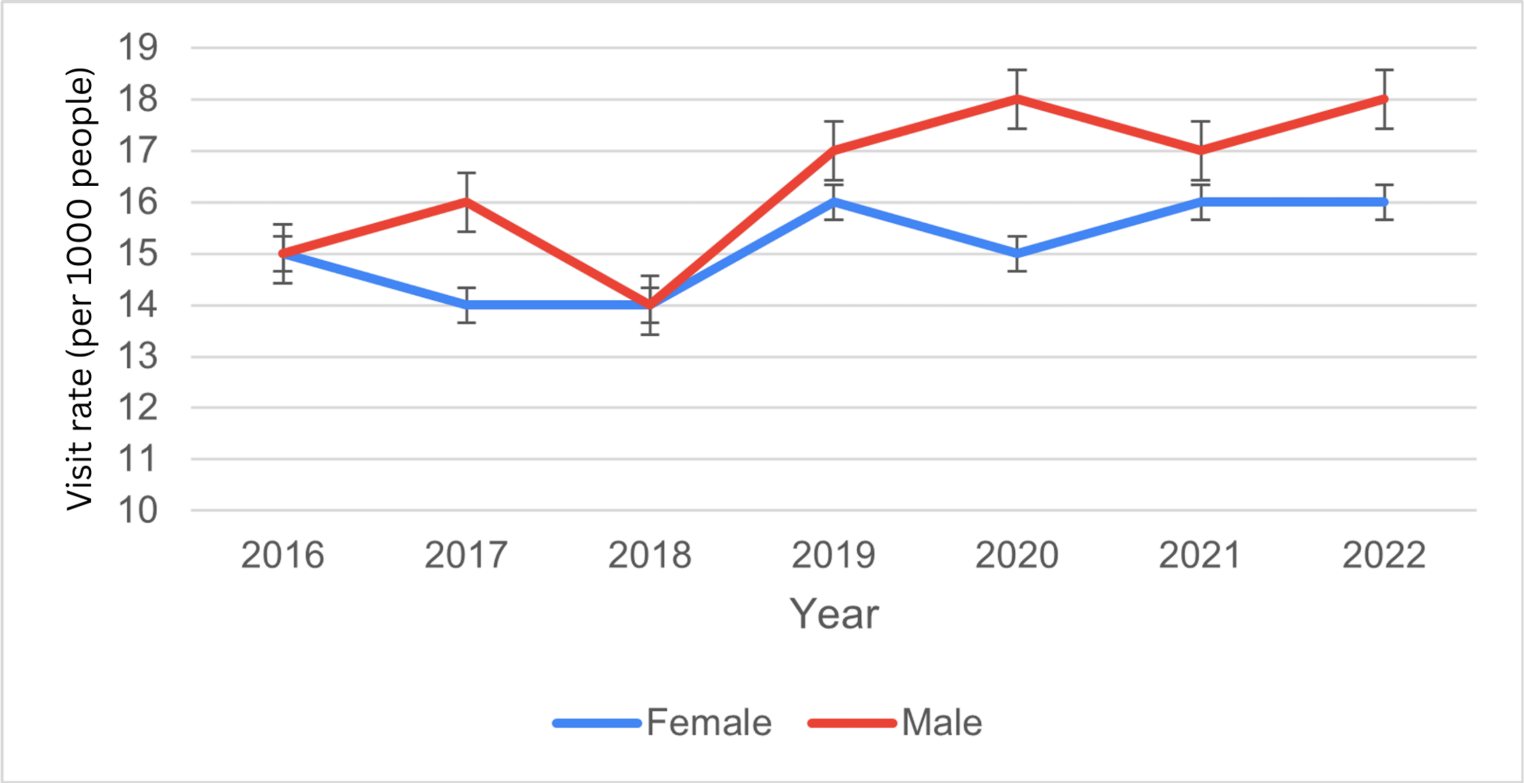
ED visit trends for circulatory system diseases by sex during the study period ED: emergency department

Among males, visits were 2.6 million in 2016 (15 per 1,000; 95% CI: 12-18), 2.8 million in 2017 (16 per 1,000; 95% CI: 11-20), 2.4 million in 2018 (14 per 1,000; 95% CI: 12-17), 3.0 million in 2019 (17 per 1,000; 95% CI: 14-21), 3.1 million in 2020 (18 per 1,000; 95% CI: 13-22), 2.9 million in 2021 (17 per 1,000; 95% CI: 13-20), and 3.0 million in 2022 (18 per 1,000; 95% CI: 14-22). These findings indicate that while visit rates fluctuate for both sexes, males consistently exhibited slightly higher rates, with peaks in 2019 and 2020 (Figure 1). The t critical two-tail value for females was 2.44, with a p-value of 0.02, indicating a statistically significant difference in visit rates. The ED visit trends for circulatory system disease by sex have been presented in Figure 1 below.

Based on age group

ED visit rates for circulatory system diseases varied significantly across age groups from 2016 to 2022. Individuals aged 18-44 years accounted for 1.6 million visits in 2016 and 2017 (5 per 1,000; 95% CI: 3-6, 4-7), increased to 2.3 million in 2018-2019 (7 per 1,000; 95% CI: 5-9), remained at 2.3 million in 2020-2021 (7 per 1,000; 95% CI: 4-10, 5-10), and declined to 1.6 million in 2022 (5 per 1,000; 95% CI: 3-6).

**Figure 2:**
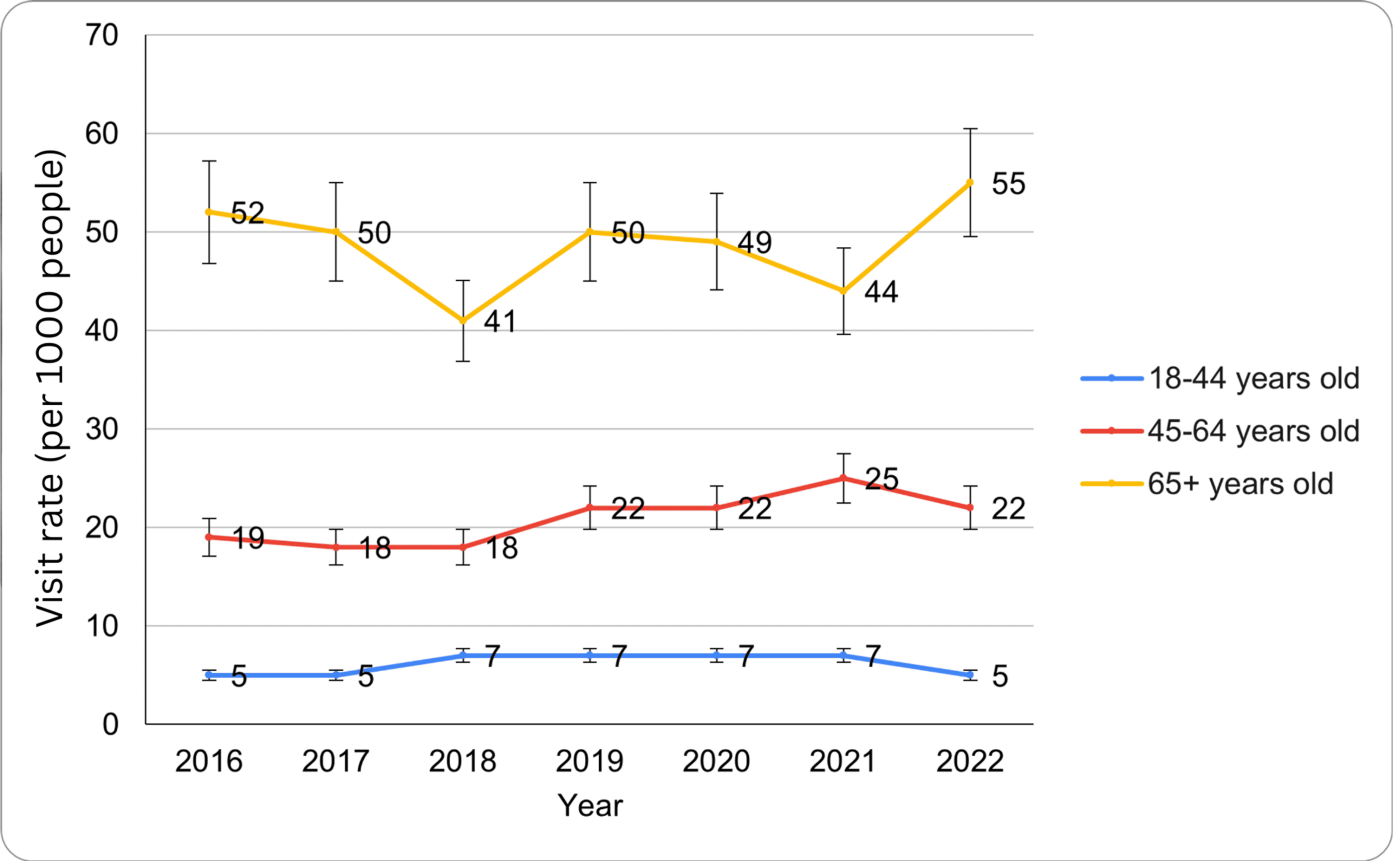
ED visit trends for circulatory system diseases by age groups during the study period ED: emergency department

Those 45-64 years had 2.2 million visits in 2016 (19 per 1,000; 95% CI: 15-23), 2.1 million in 2017-2018 (18 per 1,000; 95% CI: 13-23, 14-22), 2.6 million in 2019-2020 (22 per 1,000; 95% CI: 17-26, 15-28), peaked at 3.0 million in 2021 (25 per 1,000; 95% CI: 18-31), and then 2.6 million in 2022 (22 per 1,000; 95% CI: 16-27). Age group differences were also significant, with an F critical value of 3.88 and an F value of 300.5 (p<0.05), highlighting substantial variations among the 18-44, 45-64, and 65+ age groups. These findings indicate that ED visit rates for circulatory system diseases were consistently highest among older adults, with fluctuations observed across all age groups over the study period. The ED visit trends for circulatory system diseases by age groups have been presented in Figure 2 below.

Results by race/ethnicity

ED visit rates for circulatory system diseases varied across racial and ethnic groups from 2016 to 2022, with notable disparities. Non-Hispanic White individuals' visits are the following: 3.4 million in 2016 (17 per 1,000; 95% CI: 13-21), 3.0 million in 2017 (15 per 1,000; 95% CI: 12-19), 2.8 million in 2018 (14 per 1,000; 95% CI: 11-16), 3.6 million in 2019 (18 per 1,000; 95% CI: 15-22), 3.8 million in 2020-2021 (19 per 1,000; 95% CI: 14-24, 15-23), and 3.4 million in 2022 (17 per 1,000; 95% CI: 14-21).

**Figure 3:**
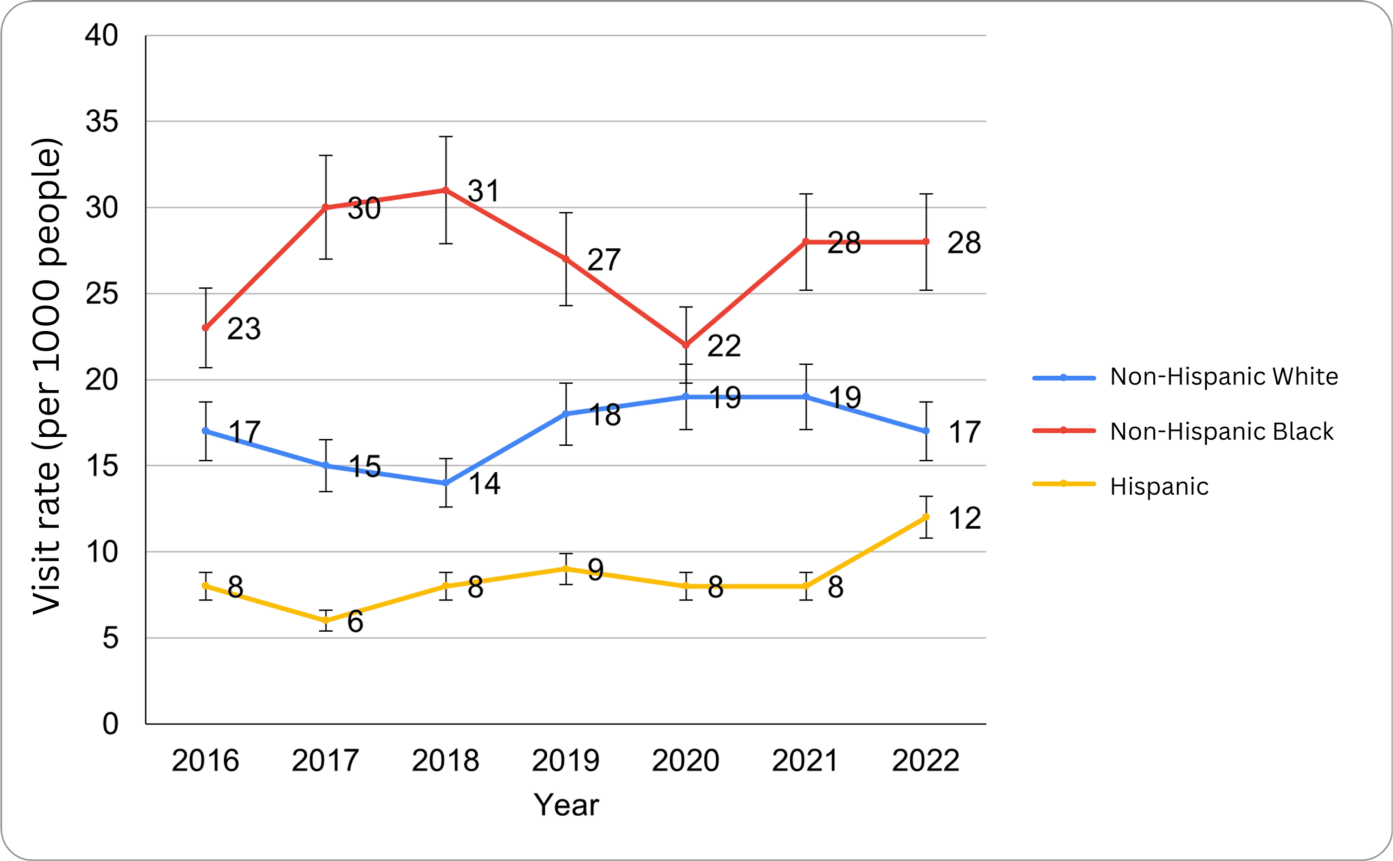
ED visit trends for circulatory system diseases by race/ethnicity during the study period ED: emergency department

Non-Hispanic Black individuals' visits are the following: 2.9 million in 2016 (23 per 1,000; 95% CI: 15-31), 3.8 million in 2017 (30 per 1,000; 95% CI: 18-42), 4.0 million in 2018 (31 per 1,000; 95% CI: 24-37), 3.4 million in 2019 (27 per 1,000), 2.8 million in 2020 (22 per 1,000), and 3.8 million in 2021-2022 (28 per 1,000; 95% CI: 19-37). Hispanic individuals' visits are the following: 1.4 million in 2016 (8 per 1,000; 95% CI: 4-11), 1.1 million in 2017 (6 per 1,000; 95% CI: 4-9), 1.4 million in 2018 (8 per 1,000; 95% CI: 4-12), 1.6 million in 2019-2021 (9 per 1,000; 95% CI: 6-13, 5-10), and 2.1 million in 2022 (12 per 1,000; 95% CI: 7-16). Similarly, racial and ethnic disparities were observed, particularly among non-Hispanic White individuals, where the F critical value was 3.88 and the F value was 82.02 (p<0.05). These findings highlight racial and ethnic disparities in ED utilization for circulatory system diseases, with non-Hispanic Black individuals consistently exhibiting the highest visit rates over the study period. The ED visit trends for circulatory system diseases by race/ethnicity have been presented in Figure 3 below.

Based on region

ED visit rates for circulatory system diseases varied regionally between 2016 and 2022. In the Northeast, there were 1.2 million visits in 2016 (17 per 1,000; 95% CI: 10-23), 0.9 million in 2018 (11 per 1,000; 95% CI: 8-15), 1.1 million in 2019 (14 per 1,000; 95% CI: 11-18), 1.4 million in 2020 (18 per 1,000; 95% CI: 9-27), 1.3 million in 2021 (16 per 1,000; 95% CI: 10-22), and 1.4 million in 2022 (18 per 1,000; 95% CI: 12-25). In the Midwest, there were 1.7 million in 2016 (19 per 1,000; 95% CI: 10-27), 1.8 million in 2017 (20 per 1,000), 1.4 million in 2018-2019 (15 per 1,000), 1.7 million in 2021 (19 per 1,000), and 1.4 million in 2022 (16 per 1,000).

In the South, there were 1.1 million in 2016 (13 per 1,000), 1.5 million in 2017 (18 per 1,000), 1.3 million in 2018 (15 per 1,000), 1.8 million in 2019 (20 per 1,000), and 1.5 million in 2022 (17 per 1,000). In the West, there were 0.8 million in 2017 (9 per 1,000), peaking at 1.4 million in 2020 and 2022 (16 per 1,000). Regional differences were noted in the Northeast, with an F critical value of 3.15 and an F value of 3.69 (p=0.03), suggesting regional variations in visit rates. These results indicate that the Midwest and Northeast had some of the highest visit rates, while the West generally reported lower rates throughout the study period. Table 2 below shows the ED visit trends for circulatory system diseases between 2016 and 2022 based on regions.

**Table 2:** ED visit trends for circulatory system diseases between 2016 and 2022 based on regions (): 95% CI CI: confidence interval, ED: emergency department

Region	2016	2017	2018	2019	2020	2021	2022	Statistical test	p-value
Northeast	17 (10-23)	-	11 (8-15)	14 (11-18)	18 (9-27)	16 (10-22)	18 (12-25)	F critical value=3.15, F value=3.69	0.03
Midwest	19 (10-27)	20 (10-27)	15 (12-27)	15	-	19 (13-24)	16 (11-22)	-	-
South	13 (9-17)	18 (12-24)	15 (12-19)	20 (16-25)	-	-	17	-	-
West	-	9	-	-	16	-	16	-	-

Based on MSA and non-MSA

ED visit rates for circulatory system diseases varied between MSAs and non-MSAs over the study period (Table 1). MSA areas recorded 4.2 million visits in 2016 (13 per 1,000; 95% CI: 11-16), 4.8 million in 2017 (15 per 1,000), 4.4 million in 2018 (14 per 1,000), and 4.7 million in 2019 (15 per 1,000). A slight decrease occurred in 2018, with a visit rate of 14 per 1,000 people (95% CI: 12-16), followed by an increase to 15 per 1,000 people in 2019 (95% CI: 12-18). Data for 2020, 2021, and 2022 were unavailable. In 2017, the visit rate was 15 per 1,000 people (95% CI: 10-21), decreasing slightly to 14 per 1,000 in 2018 (95% CI: 7-22). A sharp increase was noted in 2019, where the visit rate reached 26 per 1,000 people (95% CI: 17-35). Non-MSA areas saw data suppressed in 2016 (cell n 30), 0.7 million in 2017 (15 per 1,000), 0.6 million in 2018 (14 per 1,000), and 1.2 million in 2019 (26 per 1,000). Data for 2020, 2021, and 2022 were unavailable. MSA status did not show a significant difference, with a t-critical two-tail value of 4.3 and a p-value of 0.38. These findings suggest some fluctuations in visit rates within both MSA and non-MSA regions, with non-MSA regions showing a notable spike in 2019. Table 3 below indicates the ED visit trends for circulatory system diseases between 2016 and 2022 based on the MSA and non-MSA regions.

**Table 3:** ED visit trends for circulatory system diseases between 2016 and 2022 based on the MSA and non-MSA regions (): 95% CI CI: confidence interval, ED: emergency department, MSA: metropolitan statistical area

Region	2016	2017	2018	2019	2020	2021	2022	Statistical test	p-value
MSA	13 (11-16)	15 (11-18)	14 (12-16)	15 (12-18)	-	-	-	t critical two-tail=4.3	0.38
Non-MSA	-	15 (10-21)	14 (7-22)	26 (17-35)	-	-	-	t critical two-tail=4.3	0.38

Based on primary payment source

ED visit rates for circulatory system diseases varied by primary payment source from 2016 to 2022. For privately insured patients, rates remained relatively stable, starting at 1.0 million visits in 2016 (5 per 1,000; 95% CI: 4-7), 1.2 million in 2017 (6 per 1,000), 1.1 million in 2018 (5 per 1,000), 1.5 million in 2019 (7 per 1,000), 1.3 million in 2020 (6 per 1,000), 1.7 million in 2021 (8 per 1,000), and 1.3 million in 2022 (6 per 1,000). Rates fluctuated between five and eight per 1,000 people, peaking at eight in 2021 (95% CI: 6-11) before declining to six in 2022 (95% CI: 4-8).

**Figure 4:**
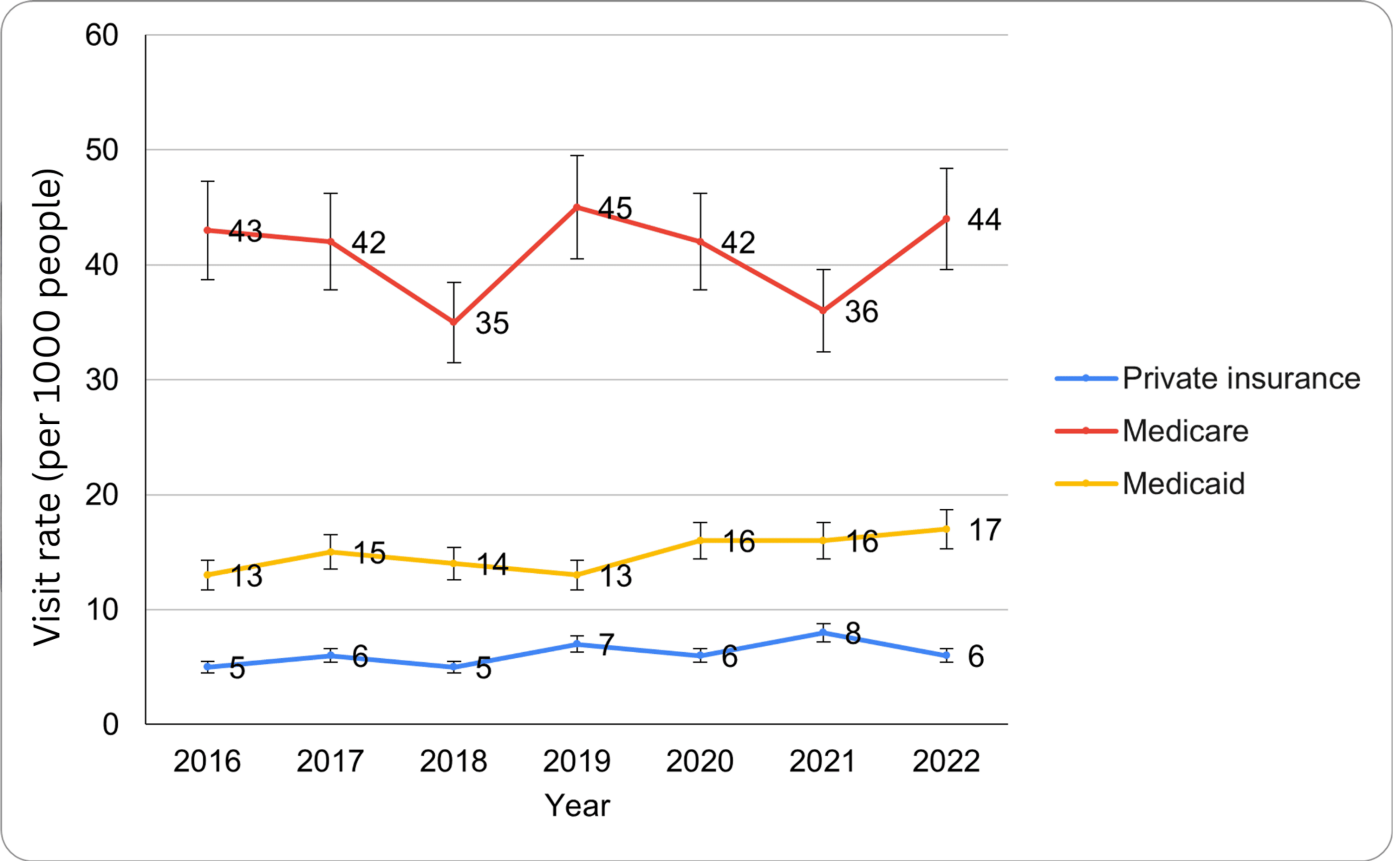
ED visit trends for circulatory system diseases by primary payment source during the study period ED: emergency department

For Medicare beneficiaries, visit rates were significantly higher, beginning at 3.5 million in 2016 (43 per 1,000), 3.4 million in 2017 (42 per 1,000), 2.8 million in 2018 (35 per 1,000), 3.6 million in 2019 (45 per 1,000), 3.4 million in 2020 (42 per 1,000), 2.9 million in 2021 (36 per 1,000), and 3.5 million in 2022 (44 per 1,000) (95% CI: 33-53). The lowest rate was 35 in 2018 (95% CI: 29-42), while the highest was 45 in 2019 (95% CI: 38-52). By 2022, the rate was 44 (95% CI: 35-54). For Medicaid patients, rates increased from 1.0 million in 2016 (13 per 1,000) (95% CI: 9-17), 1.2 million in 2017 (15 per 1,000), 1.1 million in 2018 (14 per 1,000), 1.0 million in 2019 (13 per 1,000), 1.3 million in 2020 (16 per 1,000), 1.3 million in 2021 (16 per 1,000), and 1.4 million in 2022 (17 per 1,000) (95% CI: 12-22), with fluctuations between 13 and 16 per 1,000 people from 2017 to 2021. Differences based on primary payment source were statistically significant for private insurance, with an F critical value of 3.88 and an F value of 355 (p<0.05). These findings highlight differences in ED visit rates across payment sources, with Medicare beneficiaries consistently showing the highest visit rates for circulatory system diseases. The ED visit trends for circulatory system disease by primary payment source have been presented in Figure 4 below.

## Discussion

The analysis of ED visit rates for circulatory system diseases from 2016 to 2022 highlights key trends across demographic and regional categories. Findings from this study align with prior research indicating that circulatory diseases consistently burden emergency healthcare services. A 2004 report showed that circulatory diseases were the most common cause of hospitalization in the United States, accounting for nearly 6.8 million hospital stays and $71.2 billion in costs (HCUP, AHRQ) [15]. Similarly, data from the NHAMCS have shown that ED visits for cardiovascular complaints have remained prevalent over the years due to an aging population, rising obesity rates, and persistent healthcare disparities. The stable visit rates in this study suggest that circulatory disease burdens have not diminished over time, reinforcing the need for continued healthcare resource allocation and policy interventions. The sharp decline in 2020 likely reflects ED avoidance during the early COVID‑19 pandemic, when patients deferred emergency care for fear of viral exposure and due to stay‑at‑home orders. Such avoidance may have led to delayed presentations and worsened outcomes for circulatory emergencies.

When disaggregated by sex, males and females exhibited comparable visit rates, with males showing a slight upward trend. This study found that males had higher visit rates by 2022 (18 per 1,000 people) than females (14 per 1,000). Previous studies have similarly reported higher CVD incidence in men, often attributed to biological and behavioral risk factors, including higher rates of hypertension, smoking, and obesity [16]. A study of 2023 by Rethemiotaki reported globally that CVD prevalence increased from 2010 to 2019, with females having higher rates in most conditions except ischemic heart disease, cardiomyopathy, and myocarditis, which were higher in males [17]. However, studies also suggest that women experience delays in diagnosis and treatment, which could influence their ED visit patterns. While previous research indicates that women often present with atypical cardiovascular symptoms, this study did not differentiate visit reasons by symptomatology, warranting further investigation [18].

Consistent with prior research, this study found that older adults had the highest ED visit rates for circulatory diseases. Adults aged 65+ had a visit rate of 55 per 1,000 people in 2022, significantly higher than younger age groups. This trend is well-documented in epidemiological studies, as CVD, including hypertension, myocardial infarction, and heart failure, disproportionately affects older populations [19]. The 45-64 age group had moderate visit rates, while the 18-44 age group exhibited the lowest rates. Compared to earlier studies, these findings reaffirm the strong association between age and circulatory disease prevalence, emphasizing the importance of preventive care and early interventions in middle-aged populations [20].

This study observed substantial racial and ethnic disparities in ED visit rates for circulatory diseases. Non-Hispanic Black individuals had the highest visit rates, peaking at 31 per 1,000 people in 2018 and stabilizing at 28 per 1,000 in 2022. Prior studies have similarly reported higher CVD prevalence and ED visits among Black individuals, often linked to higher rates of hypertension, diabetes, and limited access to primary care [21]. Hispanic individuals exhibited the lowest rates (8-12 per 1,000), consistent with the "Hispanic paradox," whereby protective social networks, healthier dietary patterns, and the healthy-immigrant effect may mitigate CVD risk despite socioeconomic disadvantage [22]. Nonetheless, barriers such as limited health literacy and immigration fears may also suppress ED utilization. These findings align with existing literature highlighting persistent racial and ethnic disparities in cardiovascular health outcomes and healthcare access.

This study found notable geographic differences in ED visit rates for circulatory diseases. The Midwest and Northeast had the highest rates, ranging from 15 to 20 per 1,000 people, while the West consistently reported the lowest rates (9-16 per 1,000 people). These regional variations align with previous findings indicating that CVD prevalence is highest in the Midwest and South, regions often referred to as the "stroke belt" due to their elevated rates of hypertension and obesity [23]. In contrast, lower ED visit rates in the West may reflect differences in healthcare access, preventive care measures, and lifestyle factors. The Midwest’s persistently high rates reflect entrenched "stroke-belt" risk factors, elevated hypertension, obesity, and limited rural primary care access [23,24]. In contrast, the West’s lower rates may derive from more robust preventive infrastructure, higher physical activity levels, and healthier dietary behaviors. Regional disparities in CVD burden highlight the need for targeted public health interventions to address geographic-specific risk factors.

Visit rates in MSAs remained relatively stable between 13 and 15 per 1,000 people, whereas non-MSA areas exhibited higher variability, with a peak of 26 per 1,000 people in 2019. These findings are consistent with studies showing that rural populations experience higher CVD morbidity and mortality due to limited healthcare access, fewer specialty providers, and lower socioeconomic status [24]. The higher ED visit rates in non-MSA areas may reflect an increased reliance on emergency care due to the scarcity of primary care services. This trend underscores the need for expanded healthcare infrastructure and telemedicine services in rural communities to improve CVD management and reduce preventable ED visits.

Analysis by primary payment sources revealed that Medicare beneficiaries had the highest visit rates, reaching 44 per 1,000 people in 2022, while privately insured individuals had lower rates (5-8 per 1,000 people). Medicaid recipients had moderate visit rates, increasing from 13 in 2016 to 17 per 1,000 people in 2022. These findings are consistent with prior studies indicating that Medicare recipients, primarily older adults, have the highest CVD burden and healthcare utilization rates [25]. The lower ED visit rates among privately insured individuals may reflect better access to outpatient care and preventive services. Limited data for uninsured individuals prevent robust conclusions, but existing literature suggests that uninsured patients often delay seeking care, leading to higher rates of preventable cardiovascular complications [26]. These findings highlight the role of insurance coverage in healthcare utilization and emphasize the need for policies that enhance access to primary and preventive care services [27].

The persistent burden of circulatory diseases on the ED underscores the need for targeted public health interventions. Preventive strategies, including improved management of hypertension, diabetes, and obesity, could help reduce ED visits. Expanding access to outpatient cardiovascular care, particularly in underserved regions, may mitigate the reliance on emergency services. Future research should explore the impact of healthcare policies, socioeconomic factors, and health literacy on circulatory disease-related ED visits. Additionally, longitudinal studies assessing post-pandemic trends in CVD burden could provide further insights into healthcare system adaptations and patient behaviors.

Strengths and limitations

This study provides a comprehensive analysis of ED visit rates for circulatory system diseases across a wide range of demographic and geographic factors over a seven-year period (2016-2022). The large dataset from NHAMCS ensures a robust sample size, enabling the identification of key trends and disparities in ED utilization. By examining variables such as sex, age, race/ethnicity, region, and primary payment source, the study provides nuanced insights into the factors driving ED visit rates. The longitudinal nature of the analysis allows for the detection of temporal fluctuations, including the impacts of the COVID-19 pandemic, contributing to a deeper understanding of disease burden over time.

One of the key limitations is the lack of data for certain years and categories, particularly for uninsured patients and some regions, which may introduce biases or limit the generalizability of the findings. These gaps arise from NHAMCS suppression rules for low case counts (cell n<30) and high relative standard errors, underscoring the need for cautious interpretation of subgroup trends. Additionally, while the study identifies trends and associations, it cannot establish causal relationships. Using visit rates rather than individual patient-level data limits the ability to explore patient characteristics in greater detail. Furthermore, the study does not account for other factors influencing ED visits, such as socioeconomic status, comorbidities, and healthcare infrastructure differences, which could explain the observed variations.

## Conclusions

This nationally representative analysis of NHAMCS data from 2016 to 2022 provides the first comprehensive mapping of the five most common primary circulatory diagnoses, essential hypertension, hypertensive heart or kidney disease, acute myocardial infarction, atrial fibrillation/flutter, and ischemic stroke, alongside demographic, insurance, and regional disparities and post-pandemic stabilization patterns. Our study uniquely illuminates which conditions drive the emergency burden and how utilization rebounded after the initial COVID‑19 decline by quantifying both rates and absolute ED visit counts. To translate these findings into action, we recommend evidence‑based strategies that have demonstrated effectiveness in reducing acute circulatory events and ED reliance. First, enhanced primary care outreach and blood pressure control programs, such as community health worker-led hypertension management, have reduced ED visits by up to 30% in rural settings. Second, telehealth expansion for routine cardiovascular monitoring can maintain continuity of care during public health emergencies, mitigating the 2020 care gap. Third, targeted preventive interventions in the Midwest “stroke belt,” including mobile clinics and lifestyle modification campaigns, can address region‑specific risk factors like obesity and uncontrolled hypertension. Finally, addressing insurance-related barriers, for example, streamlining Medicaid enrollment and reducing copayments, may lower avoidable ED use among underinsured groups. Our study’s novel contribution lies in its simultaneous evaluation of diagnostic spectrum, temporal trends, and subgroup differences within one robust analysis. These insights can inform policymakers and healthcare systems seeking to allocate resources, tailor community outreach, and strengthen preventive care pathways, ultimately reducing the acute circulatory disease burden on emergency services.
